# Taxonomic and functional diversity change is scale dependent

**DOI:** 10.1038/s41467-018-04889-z

**Published:** 2018-07-02

**Authors:** Marta A. Jarzyna, Walter Jetz

**Affiliations:** 1 Department of Ecology and Evolutionary Biology, Yale University, New Haven, CT 06520 USA; 2 Department of Life Sciences, Imperial College London, Silwood Park Campus, Buckhurst Road, Ascot, Berks SL5 7PY UK

## Abstract

Estimates of recent biodiversity change remain inconsistent, debated, and infrequently assessed for their functional implications. Here, we report that spatial scale and type of biodiversity measurement influence evidence of temporal biodiversity change. We show a pervasive scale dependence of temporal trends in taxonomic (TD) and functional (FD) diversity for an ~50-year record of avian assemblages from North American Breeding Bird Survey and a record of global extinctions. Average TD and FD increased at all but the global scale. Change in TD exceeded change in FD toward large scales, signaling functional resilience. Assemblage temporal dissimilarity and turnover (replacement of species or functions) declined, while nestedness (tendency of assemblages to be subsets of one another) increased with scale. Patterns of FD change varied strongly among diet and foraging guilds. We suggest that monitoring, policy, and conservation require a scale-explicit framework to account for the pervasive effect that scale has on perceived biodiversity change.

## Abstract (web summary)

The evidence for and implications of biodiversity change remain widely debated. Jarzyna and Jetz demonstrate a strong and varying scale dependence of avian taxonomic and functional diversity, highlighting the importance of scale when assessing biodiversity change.

## Introduction

Biodiversity and its many functions are undergoing rapid changes worldwide, with multifarious potential consequences for human well-being^1,2^. An appropriate measurement of this change is key to detecting the signature of anthropogenic impacts, evaluating the implications of biodiversity loss to humans, and informing monitoring and conservation programs^2,3^. Nevertheless, evidence of local biodiversity change often remains complex and contradictory^4,5^. For example, recent studies^6,7^, albeit contested^8^, claimed a lack of systematic local biodiversity loss across multiple taxa, regions, and realms. Notably, that work^6,7^ pooled data from locations differing up to eight orders of magnitude in area. Despite the suggestion that spatial scale may influence evidence of biodiversity change^9,10^, to date, a quantitative assessment of these issues is lacking (but see refs. ^11–13^).

Taxonomic diversity (species richness (TD)) remains the main measure of biodiversity despite the recognition that it does not account for the many different ecological functions^14,15^ of species comprising communities and may thus not account for the implications of biodiversity change for the functioning of ecosystems and their services for humans^5,16^. Losses or gains of some species might have much greater functional implications for ecosystems than those of others^17^ and differently affect assemblage functional diversity^18^. Acknowledging species’ functional attributes is seen as vital for understanding the processes responsible for the spatial and temporal dynamics of species occurrence and community assembly^19–21^, and is increasingly considered crucial for conservation prioritization^22^. Few studies, however, have placed functional diversity (FD) change in a scale-dependent context.

Here, we use uniquely suited, detection-corrected^23^, and near-continental data from the North American Breeding Bird Survey (BBS) from a nearly 50-year period, and extended to extinctions at the global scale^24^, to demonstrate a pervasive scale and metric dependence of taxonomic and functional diversity change. We find that TD and FD increased at all but the global scale, though change in TD exceeded change in FD toward large scales, suggesting strong trait redundancy at those scales. Patterns of change in functional diversity varied strongly among diet and foraging guilds, raising concerns about the loss of critical ecosystem functions. Our results indicate that scale-explicit framework should be adopted in monitoring, policy, and conservation to account for the pervasive effect that scale has on perceived biodiversity change.

## Results

### Taxonomic diversity

**Fig. 1:**
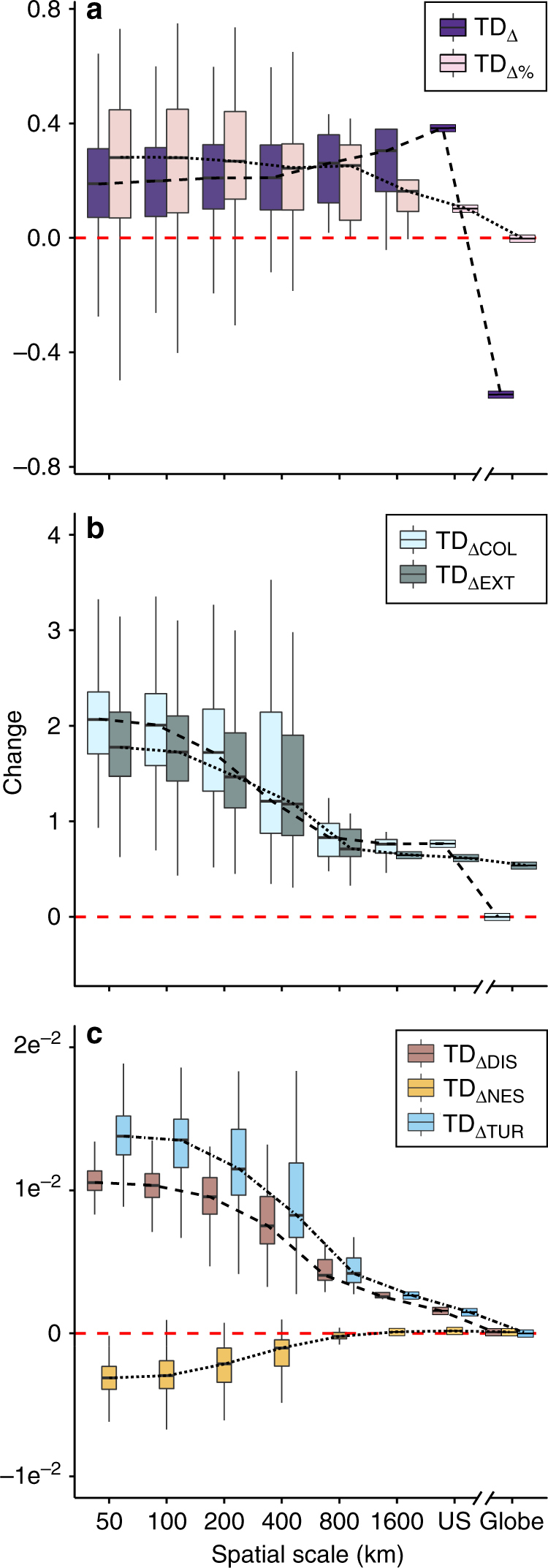
Changes in avian taxonomic diversity are scale dependent. Scale dependence is evident in the fitted slopes between time and **a** taxonomic diversity (TD_Δ_) and relative change in taxonomic diversity (TD_Δ%_) and **b** the number of colonizations and extinctions (TD_ΔCOL_ and TD_ΔEXT_, respectively). **c** Changes in temporal dissimilarity of avian taxonomic diversity (TD_DIS_) given by the fitted slopes between TD_DIS_ and time (TD_ΔDIS_) also show strong scale dependence, as do the components of TD_ΔDIS_, temporal nestedness, and turnover (TD_ΔNES_ and TD_ΔTUR_, respectively). Boxes represent the 25th and 75th percentiles, lines within the boxes represent the 50th percentile (median), and whiskers represent 2.5th and 97.5th percentiles. For the continent-wide analysis, bird occurrence records were obtained from the North American Breeding Bird Survey (1969–2013). We included 494 species, excluding nocturnal, crepuscular, and pelagic species. For the global analysis, we used data of global extinctions from ref. ^24^

**Fig. 2:**
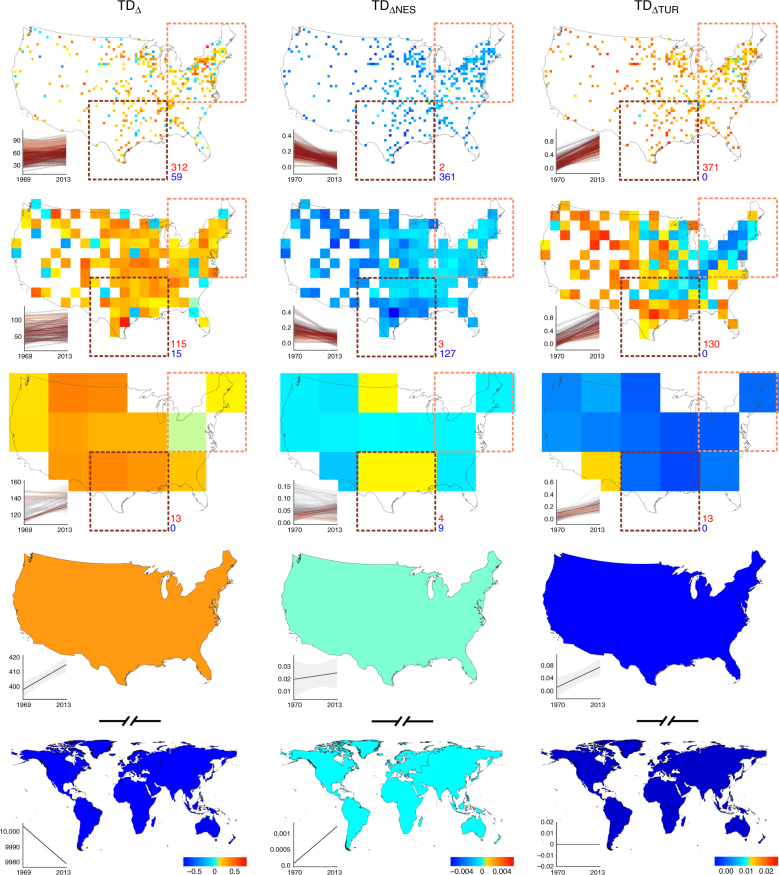
Spatial variation in avian diversity change across different spatial scales. The scales shown, from top to bottom, are 50 km, 200 km, 800 km, the continental United States, and the globe. Fitted positive and negative slopes (see main text) indicate increases and declines in taxonomic diversity (TD_Δ_, left panel), temporal nestedness of taxonomic diversity (TD_ΔNES_, middle panel), and temporal turnover of taxonomic diversity (TD_ΔTUR_, right panel). Red and blue numbers indicate the number of grid cells for which these trends were positive and negative, respectively. Insets show the fitted slopes between the respective measure of change (*y*-axis) and time (*x*-axis; calendar year) for the cell inside the brown (brown lines) or beige (beige lines) quadratic regions. For data, see Fig. 1

Across spatial scales from 50 km (here, referred to as local scale) to the continental United States (here, referred to as the continental scale), and the entire globe (21 global extinctions since 1969; Supplementary Table 1^24^), we evaluated detection-corrected^23^ change in avian TD (TD_∆_) and relative change in avian TD (TD_∆%_) for the years 1969–2013. We also evaluated the number of species colonizations (TD_COL_) and extinctions (TD_EXT_) and their temporal change—TD_∆COL_ and TD_∆EXT_—as the fitted slopes with time (see Methods). An unbiased temporal coverage was obtained by retaining only those BBS routes that were surveyed both in 1969 and 2013 time periods (see Methods). TD_∆_ and TD_∆%_ were positive (i.e., species richness increased across time) and relatively constant across spatial scales up to regional (1600 km) and continental scales, where TD_∆_ increased and TD_∆%_ declined (Figs. 1a and 2). These changes were underpinned by an increase in the number of colonizations (positive TD_∆COL_) and extinctions (positive TD_∆EXT_) across time at local scales followed by still positive but declining TD_∆COL_ and TD_∆EXT_ towards coarser scales (Fig. 1b, Supplementary Fig. 1). The rate of decline in TD_ΔCOL_ and TD_∆EXT_ with area increased as the scale approached the regional scales and then stabilized towards the continental domain (Fig. 1b). At all but the global scale, TD_∆COL_ exceeded TD_∆EXT_; from the regional scale toward the globe, TD_∆COL_ declined to ultimately zero which, together with a non-zero global TD_∆EXT_, resulted in a switch to a distinctly negative TD_∆_ at the global scale.

At local scales of our analysis (50 km), increases in species richness strongly dominated (~90%), but with significant regional variation (Fig. 2, Supplementary Fig. 2). The northeastern United States saw both declines and gains in bird richness at the local scale, resulting in overall declines in species richness at the regional scale (Fig. 2, Supplementary Fig. 2). The southeastern United States saw declines in bird species richness at local, intermediate (100–800 km), and regional scales, while the western United States saw both gains and declines (Fig. 2, Supplementary Fig. 2). At the local and intermediate scales, increases in species richness are likely a result of new habitats as part of ongoing land cover and climate change^25^ and restoration efforts^26^, and introduction of exotic species^27^. Species’ range shifts following anthropogenic climate change^28^ or introduction of species from other biogeographic regions^27^ likely contributed to species gains at regional and continental scales.

### Temporal dissimilarity, nestedness, and turnover in taxonomic diversity

Capturing temporal shifts in assemblage composition, such as those expected to follow environmental and biotic change, provides a more sensitive indicator of community change^7^. For each spatial scale, we quantified temporal dissimilarity (i.e., temporal beta diversity) in community composition as $\mathrm {TD}_{\mathrm {DIS}} = \frac{{\mathrm {TD}_{\mathrm {COL}} + \mathrm {TD}_{\mathrm {EXT}}}}{{2\mathrm {TD}_{\mathrm {PERS}} + \mathrm {TD}_{\mathrm {COL}} + \mathrm {TD}_{\mathrm {EXT}}}}$, where TD_PERS_ is the number of species that persisted through time (see Methods), and measured its temporal trend, TD_∆DIS_, as the fitted slopes with time. We find that TD_ΔDIS_, though positive at all spatial scales, declined with coarsening spatial scale (Fig. 1c). The rate of decline in TD_ΔDIS_ with area increased as the focal cell area approached the continental domain and TD_∆COL_ and TD_∆EXT_ declined sharply.

Temporal dissimilarity has two components with different implications, temporal nestedness (the tendency of assemblages to be subsets of one another^29^; TD_NES_) and temporal turnover (the replacement of species over time; TD_TUR_)^30^. For example, an assemblage that over time gains new species without losing any is fully temporally nested, whereas temporal turnover dominates if species gains are matched with losses—with any intermediate scenarios possible. While turnover is often a consequence of neutral dynamics or environmental sorting^30^, nestedness reflects a non-random process of species loss or gain as a consequence of any factor that promotes species filtering^30^.

As expected given their complementarity^30^, temporal nestedness and turnover showed opposite scaling characteristics (Fig. 1c, Supplementary Fig. 3). Assemblages separated by longer time periods were less nested within one another (negative TD_ΔNES_) at local to intermediate scales, but TD_ΔNES_ became slightly positive toward regional and continental scales (Fig. 1c). TD_TUR_ increased for sites separated by longer time periods (positive TD_ΔTUR_) at all scales; however, that increase declined with spatial scale and reached zero at the global level (Fig. 1c). Such scale dependence suggests that continual turnover in assemblage composition resulting from neutral dynamics generally dominates at local scales^31^, while deterministic factors promoting species filtering across time are more pronounced at regional and continental than local levels. The scale dependence of nestedness and turnover corresponded closely to that of colonizations and extinctions, potentially suggesting that strong nestedness at coarse spatiotemporal scales is a direct result of the colonizations not being offset by the equivalent number of extinctions (the globe excepted). Our findings give credence to the idea of no ecological bounds to biodiversity^32^, although, and perhaps critically, changes in resource availability^33^ or potential extinction debt^34^ were not assessed here. As we move from the scale of potentially interacting species and local communities to that of an entire region or continent, the lack of ecological bounds becomes more pronounced, as also argued by others^32^.

### Functional diversity

The relationship between functional diversity change (FD_Δ_) and TD_Δ_ depends on the clustering vs. overdispersion of traits or, in an alternative view, the level of species functional distinctness (i.e., the uniqueness of species in terms of their trait-based position) relative to other species in an assemblage^23^. Assemblages with low mean species functional distinctness (i.e., high trait redundancy) might see |FD_Δ_|<|TD_Δ_| because gains and losses will mostly affect redundant traits. Conversely, we expect |FD_Δ_|>|TD_Δ_| for assemblages with high mean species functional distinctness (trait overdispersion) as change will more readily invoke unique traits. Biotic processes, such as competition, are expected to facilitate functional distinctness and presumed strongest at local scales^35,36^, while trait clustering and redundancy are expected to prevail at coarser scales with higher species richness^37^ and stronger environmental filtering^38^. We therefore expect |FD_Δ_|>|TD_Δ_| at local scales and the reverse (|FD_Δ_|<|TD_Δ_|) at regional and continental scales.

**Fig. 3:**
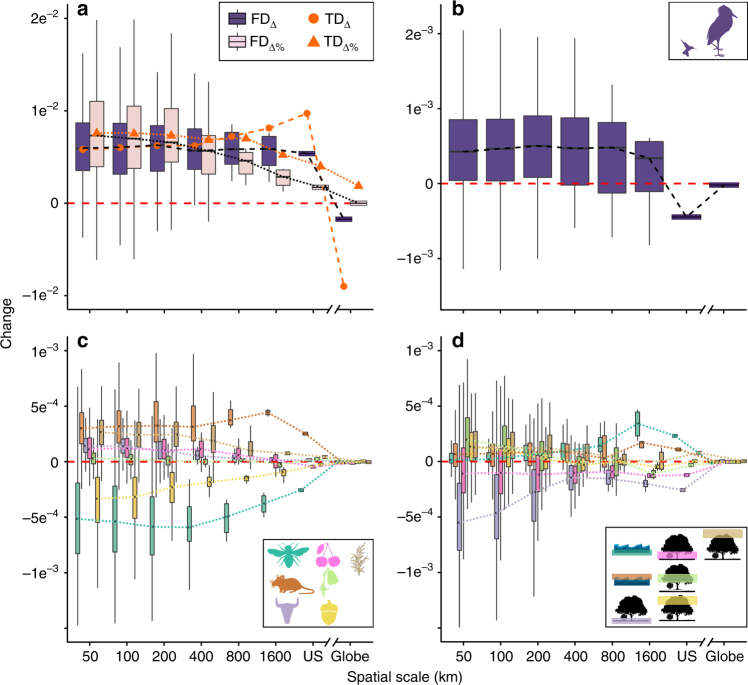
Scale dependence of changes in avian functional diversity and functional trait space. Changes were calculated as the fitted slopes between time and **a** functional diversity (FD_Δ_) and relative change in functional diversity (FD_Δ%_), **b** assemblage mean body mass, **c** proportions of different diets, and **d** proportions of different foraging niches. Diet and foraging niche categories consisted of seven axes each: proportions of invertebrates, vertebrates, carrion, fresh fruits, nectar and pollen, seeds, and other plant materials in species’ diet (diet category), proportional use of water below surface, water around surface, terrestrial ground level, understory, mid canopy, upper canopy, and aerial (foraging niche category). To facilitate the visual comparison, FD_Δ_ was standardized to fit the scale of the other measures. Compilation of function-relevant traits was obtained from ref. ^71^. For other details see Fig. 1

We find that scaling of FD_Δ_ and TD_Δ_ followed each other closely at local and intermediate spatial scales, but |FD_Δ_| was increasingly exceeded by |TD_Δ_| toward coarser scales (Fig. 3a, Supplementary Figs. 4–5), suggesting—as expected—strong trait clustering and redundancy at those scales. This implies that, at very large scales (i.e., regional, continental, and global), increases or declines in species richness are no longer associated with the addition or loss of unique ecological functions. We hypothesize that |FD_Δ_|<|TD_Δ_| even at finest analyzed scale (50 km) may be due to the fact that spatial dynamics may play out at yet finer scales of single territories, where biotic interactions such as interspecific competition are expected to strongly influence community assembly.

We found that this general picture of FD_Δ_ scale dependence varies strongly among functional components. For each year and spatial scale, we quantified the temporal change in mean body mass and also in the relative prevalence of diet and foraging niches (measured as their respective proportion across all species in an assemblage; see Methods). Changes in mean assemblage body mass overall mimicked those of FD_Δ_ (Fig. 3b), though continental scale saw declines in mean body mass despite increases in FD. Increases in mean assemblage body mass at all but continental scale suggest that colonizing species tend to be on average larger than resident species and/or that the probability of local extinction is negatively related to species’ body mass^39^. At the continental scale, a decline in mean assemblage body mass might be a response to changing climatic conditions^40^. As climate warms, smaller species are expected to lose the disadvantage associated with increased heat loss, resulting in increases in relative proportion of smaller species^41^.

Significantly, the individual diet and foraging guilds behaved very heterogeneously across scales (Fig. 3c, d). Raptors increased in their relative prevalence (i.e., proportional richness) at all spatial scales (Fig. 3c, Supplementary Fig. 7), potentially as a result of the dichlorodiphenyltrichloroethane (DDT) ban in 1972^42^. Scavengers or birds feeding on plant matter increased in their assemblage prevalence at local and intermediate scales, but stagnated toward coarser scales (Fig. 3c, Supplementary Fig. 7). In contrast, insectivores and ground-foragers consistently decreased in relative prevalence across all spatial scales, raising concerns about the loss of their critical functions (e.g., pest-controlling; Fig. 3c, d, Supplementary Figs. 7 and 8). Several factors might have contributed to declines in insectivorous and ground-foraging birds, including the use of neonicotinoid pesticides^43^, the documented dramatic declines in insect abundances^44,45^, or climate change-caused trophic mismatches^46,47^. Yet, other groups such as open water foragers showed little change locally, but strong increases toward regional and continental spatial scales, potentially reflective of increased habitat availability at that scale combined with strong dispersal ability (Fig. 3d, Supplementary Fig. 8). Most functional components showed little or no temporal change at regional and continental scales (Fig. 3, Supplementary Figs. 7 and 8), further suggesting increasing functional redundancy and, potentially, resilience with coarsening spatial scale. These findings add an important scale context to previous work on diet breadth^48^ and guild^49^ or trait correlates of observed or projected^50^ biodiversity change (see also ref. ^51^) and highlight the complexity of the interaction between scale and functional consequences of biodiversity change.

## Discussion

Our findings highlight the need for a better understanding of the uncovered scaling patterns for other parts of the tree of life. Select evidence hints at potentially varying scale dependence of extinction events in birds, plants, and butterflies^11^. Due to the strong effects of dispersal limitation on range expansion we also expect likely pronounced cross-taxon differences for colonization^52^: as species expand their range, gains are likely to occur quickly at local scales before the signal spreads to larger scales^53^, but this may be different for strong dispersers such as birds (but see ref. ^54^). Additional research is needed to capture the fundamental scale dependence of biodiversity change for more taxa.

Maintaining the functioning of ecosystems and halting biodiversity loss are at the core of several targets of the United Nations’ Convention on Biological Diversity and the Sustainable Development Goals, and their appropriate evaluation is a focus of the Intergovernmental Science-Policy Platform on Biodiversity and Ecosystem Services (http://www.ipbes.net). While not overtly raised, scale is implicit in the policy, management, and monitoring activities relevant to all targets. Our findings demonstrate how scale affects both evidence and implications of biodiversity change. Gains or apparent stasis at one scale may be fully reconcilable with losses at others, their functional implications will vary by scale and functional component, and both the detection and management of biodiversity change may need to be reconciled with the spatial and temporal scale most relevant to the question. This conclusion lends credence to the need for a cross-scale integration of biodiversity change evidence through the support of models and remote-sensing^55^, as well as a stronger consideration of the functional dimensions of change^17^. Biodiversity monitoring, policy, and conservation will likely benefit from advancing and adopting a decidedly scale-explicit framework.

## Methods

### Data

To evaluate changes in avian diversity across spatial and temporal scales, we used data from the North American Breeding Bird Survey (BBS, http://www.pwrc.usgs.gov/), an avian monitoring program established in 1966 to track the status and trends of bird populations^56^. BBS data are collected annually during the height of the avian breeding season along over 4100 survey routes located across North America, making it the most comprehensive avian survey program in the United States and probably worldwide^57^. Each survey route is approximately 40 km long and contains five segments and 50 stops at approximately 800 m intervals^58^. At each stop, observers conduct a 3 min point count during which every bird observed or heard within an approximately 400 m radius is recorded. Data collected prior to 1995 are available for each segment of a route, but not for each point. The BBS follows a standardized monitoring protocol, allowing for sound comparison of avian diversity patterns through time.

We excluded data from 1966 to 1968 because of the limited spatial coverage at the inception of the program. To reduce spatial sampling bias and improve representation of all US regions^59^, we conducted spatial subsampling using Bird Conservation Regions (BCRs) (http://nabci-us.org/resources/bird-conservation-regions/). We removed routes from BCRs with more than 30 routes (in order of proximity to remaining routes) until all BCRs had only 30 or fewer routes. While this subsampling did not achieve a fully spatially balanced sample (the eastern United States retained more BBS routes than the western United States), we were able to attain reasonably unbiased spatial coverage without compromising the sample size. To obtain an unbiased temporal coverage, we retained only those BBS routes that were surveyed both in 1969 and 2013 time periods, bringing the total number of BBS routes retained in this analysis to 447. We characterized each route for its median latitude and longitude, and also for its elevation (ELEV) based on the National Elevation Dataset (http://ned.usgs.gov/) averaged over each 1 km pixel intersecting the route. The shapefile with the BBS route trajectory was retrieved from the United States Geological Survey (USGS) Patuxent Wildlife Research Center via a spatial data repository (https://geo.nyu.edu/catalog/stanford-vy474dv5024).

Following others^60^, we removed from all routes records for species that were left unidentified or are generally poorly captured by the BBS survey methodology (i.e., nocturnal and crepuscular species, pelagic species), resulting in 494 species analyzed. Because BBS resulting estimates of abundance might under certain circumstances be less reliable than estimates of occurrence^61^ and because using abundance data would violate the closure assumption thus precluding the use of N-mixture modeling framework (where closure is assumed over individuals, not species), we used presence–absence data.

To estimate change in avian diversity across the entire globe, we consulted the evaluation of ref. ^24^, from which we identified species that have gone extinct since 1969. We considered species designated as Extinct, Extinct in the Wild, and Critically Endangered (Possibly Extinct) (Supplementary Table 1).

### Multispecies occupancy modeling

Ignoring species’ imperfect detection in the evaluation of biodiversity dynamics might cause erroneous inference^4,23^. We used multispecies occupancy models^62,63^ to account for species imperfect detection in the estimates of taxonomic and functional diversity^23^. In order to discern a non-detection from a point-level absence at each location, occupancy modeling techniques rely on the repeated sampling protocol^64^. Because the BBS monitoring program does not follow the repeated sampling protocol (i.e., each BBS survey route is visited only once during each breeding season), the five segments falling within each BBS route represented “repeated samples” characterizing the route^65–67^. Space-for-time substitution is often used in occupancy modeling when temporal replicates are not available^65,68,69^.

In order to account for imperfect detection across the entire time series, we ran a total of 45 multispecies occupancy models (i.e., one for each year, 1969–2013). Observed data, *y*_*i,j,k*_, for species *i* = 1, 2,…, 494, at site *j* = 1, 2,…, *j*, on sampling segment *k* = 1, 2,…, 5, were modeled as resulting from the imperfect observation of a true occurrence state, *z*_*i,j*_, given a probability of detection, *p*_*i*,*j*,*k*_ . Because not all BBS routes were monitored each year, *j* varied among years. This observation process was modeled as the Bernoulli random variable *y*_*i,j,k*_ ~ Bern(*p*_*i,j,k*_ · *z*_*i,j*_), where *z*_*i,j*_ = 1 if species *i* was truly present at site *j*, and *z*_*i,j*_ = 0 if species *i* was absent at site *j*. The true occurrence state was specified as *z*_*i,j*_, ~ Bern(*ψ*_*i,j*_), where *ψ*_*i,j*_ was the probability of occurrence of species *i* at site *j*. We estimated probabilities of occurrence for undetected species using data augmentation following Kéry and Royle^62^. The resulting estimates of probability of species occurrence *ψ*_*i,j*_ provided an indication of the likelihood of species presence given it went undetected.

We modeled probability of occurrence as a linear function of ELEV as follows: ${\mathrm {logit}}( {\psi _{i,j}} ) = \beta _{0,i} + \beta _{1,i}\cdot {\mathrm {ELEV}}_{\mathrm {j}}$. Probability of detection was modeled as an intercept only as follows: ${\mathrm {logit}}( {p_{i,j}} ) = \alpha _{0,i}$, because measurements of potential detection covariates (e.g., weather conditions, time of survey, etc.) were not available for each segment of the route. To estimate model fit, we computed posterior predictive *p* values. Posterior predictive (Bayesian) *p* values allow model evaluation based on comparing the distribution of random draws of new data generated using parameters from the model fitted to the observed data^70^. If a model is a good fit to the data, then the replicated data predicted from that model should look similar to the observed data^70^ and the ratio of their posterior distributions be close to 1. Posterior predictive *p* values of ca. 0.5 further indicate good model fit. For all models in our analysis, the posterior predictive *p* values were equal to approximately 0.5 and the ratio of posterior distributions of new and fitted data close to 1 (see Supplementary Fig. 9). R code is available in the Supplementary Note 1.

Each species was fit to all detection and occurrence parameters. To avoid instances where probability of occurrence *ψ*_*i,j* _> 0 for species that are unlikely to be present given their ecological constraints, we further constrained the model so that only species detected within a given BCR in that year could have *ψ*_*i,j* _> 0 at a route located within that BCR. To ensure that this assumption did not bias the results of the study, we compared probabilities of species’ occurrence constrained by this assumption with unconstrained probabilities of species’ occurrence (see Supplementary Fig. 10). We estimated model parameters using Bayesian analysis, using program JAGS (Just Another Gibbs Sampler; http://mcmc-jags.sourceforge.net/) via R (version 3.2.3; https://www.r-project.org/) using the package rjags (https://cran.r-project.org/web/packages/rjags/index.html).

### Taxonomic and functional diversity

We considered eight consecutive equal area spatial scales: 50 km, 100 km, 200 km, 400 km, 800 km, 1600 km, the continental United States, and the globe. At each scale (with exception of the globe), the probability of a species occurrence in a given grid cell was computed by taking the maximum value of probabilities of occurrence of that species across BBS routes falling, in their majority, within that grid cell. At each time period, we retained a given grid cell for the analysis of taxonomic and functional diversity (and change therein) only if it contained all BBS routes initially falling within its bounds—e.g., if a given grid cell contained a total of 10 BBS routes, it would be removed from the analysis at any time period when the number of routes was <10. This ensured that the number of routes contributing to a given grid cell remained constant across time. For the globe, we considered all non-extinct species to be present.

For each grid cell and year between 1969 and 2013, we calculated detection-corrected taxonomic (TD) and functional (FD) diversity. For each grid cell at a given spatial grain, TD was given as summed probability of species occurrence: ${\mathrm {TD}}_{\mathrm {j}} = \mathop {\sum}\nolimits_{i = 1}^{494} {\psi _{i,j}}$. We based estimates of functional diversity on a compilation of function-relevant traits in Wilman et al.^71^ and following ref. ^23^. Three trait categories were included: body mass, diet, and foraging niche. The diet and foraging niche categories included seven axes each: proportions of invertebrates, vertebrates, carrion, fresh fruits, nectar and pollen, seeds, and other plant materials in species’ diet (diet category); proportional use of water below surface, water around surface, terrestrial ground level, understory, mid canopy, upper canopy, and aerial (foraging niche category). For the extinct species whose trait information was not included in ref. ^71^, we used trait values for sister species and/or conducted the literature search for a species’ ecological preferences (Supplementary Table 1). Following existing practice^50,72,73^ we calculated multivariate trait dissimilarity using Gower’s distance for each pairwise combination of the 494 species in the dataset. Equal weights were given to each of the trait categories and to each axis within the trait categories (i.e., each diet and foraging niche variable was given a 1/7 weight, whereas the weight of body mass was 1). As distance metric we used Gower’s distance, because this index can handle quantitative, semi-quantitative, and qualitative variables and assign different weights to individual traits^73^. The functional dendrogram was then built using UPGMA (Unweighted Pair Group Method with Arithmetic Mean) clustering. UPGMA clustering has the highest cophenetic correlation coefficient among most popular clustering methods (Ward, Single, Complete, WPGMA, WPGMC, and UPGMC clustering methods)^50^ and the lowest 2-norm index^74^, ensuring most faithful preservation of the original distances in the dissimilarity matrix.

For each grid cell and spatial scale, the master functional dendrogram was pruned of branches for species whose *ψ* = 0. The branch lengths of each species in the remaining functional dendrogram were then weighted by the probability of species *i*’s occurrence at that BBS route, *ψ*_*i*_, as follows: all terminal branches were multiplied by *ψ*_*i*_ and all intermediate branches were given the weight $w = 1 - \mathop {\prod}\nolimits_{i \in I} {\left( {1 - \psi _i} \right)}$, where *I* represents all species included in the node of the intermediate branch^50^.

### Temporal change in avian diversity

We quantified change in avian diversity through time with two general measures: temporal change in α diversity and change in temporal β diversity. Both α and temporal β diversity were assessed for taxonomic and functional diversity. To measure temporal change in α diversity, we calculated, for each spatial scale, the slope of the long-term relationship between TD|FD and time (TD_Δ_|FD_Δ_). We also calculated the slope of the long-term relationship between TD|FD relative to the first year of sampling and time (TD_Δ%_|FD_Δ%_).

To measure change in assemblage composition through time, we quantified change in temporal β diversity (temporal dissimilarity). Temporal dissimilarity quantifies differences in species composition between two (or more) samples separated in time. Because dissimilarity incorporates shifts in community composition, it potentially provides a more sensitive indicator of community change than α diversity^7^. We used Sørensen dissimilarity index between ensuing year and the first year of the dataset as a measure of temporal dissimilarity of taxonomic (TD_DIS_) and functional (FD_DIS_) diversity. TD_DIS_ between ensuing year *m* and 1969 (i.e., the first year of the dataset) was calculated as $\mathrm {TD}_{\mathrm {DISm}} = \frac{{b + c}}{{2a + b + c}}$, where *a* are the species that persisted between years 1969 and *m*, *b* are the sum of the species that colonized (for the first time) the site between years 1969 and *m*, and *c* are the sum of the species that went locally extinct between years 1969 and *m* and never recolonized. We calculated *a* by summing up the product of probabilities of species occurrence in year *m* and 1969 (*ψ*_*m*_ and *ψ*_1969_, respectively). We calculated *b* as $\mathop {\sum}\nolimits_{1970}^m {\psi _m\left( {1 - \psi _{m - 1}} \right)}$ for only those species whose *ψ* in years between 1969 and *m* − 1 never exceeded 0.8 (to ensure counting only first colonizations). We calculated *c* as $\mathop {\sum}\nolimits_{1970}^m {\psi _{m - 1}\left( {1 - \psi _m} \right)}$ for only those species whose *ψ* in years from *m* + 1 until 2013 never exceeded 0.8 (to ensure that these species never recolonized). To estimate the FD_DIS_, we also used Sørensen dissimilarity index^75^ but replaced TD with FD^50^. FD_DIS_ was calculated as$\mathrm {FD}_{\mathrm {DISm}} = \frac{{e + f}}{{2d + e + f}}$, where *d* is the FD of *a*, *e* is the FD of *b*, and *f* is the FD of *c*.

Because two different phenomena (i.e., nestedness and species replacement, often termed turnover) can produce differences in species composition between two sites, we quantified these two components of dissimilarity—i.e., nestedness (TD_NES_|FD_NES_) and turnover (TD_TUR_|FD_TUR_) for both taxonomic and functional diversity. TD_NES_ was measured as nestedness-resultant component of Sørensen dissimilarity and given as $\mathrm {TD}_{\mathrm {NESm}} = \frac{{\max \left( {b,c} \right) - {\mathrm{min}}\left( {b,c} \right)}}{{2a + b + c}}\cdot \frac{a}{{a + {\mathrm{min}}\left( {b,c} \right)}}$. TD_TUR_ between ensuing year *m* and 1969 was quantified as $\mathrm {TD}_{\mathrm {TURm}} = \frac{{{\mathrm{min}}\left( {b,c} \right)}}{{a + {\mathrm{min}}\left( {b,c} \right)}}$. FD_NES_ and FD_TUR_ were calculated as $\mathrm {FD}_{\mathrm {NESm}} = \frac{{\max \left( {e,f} \right) - {\mathrm{min}}\left( {e,f} \right)}}{{2d + e + f}}\cdot \frac{d}{{d + {\mathrm{min}}\left( {e,f} \right)}}$ and $\mathrm {FD}_{\mathrm {TURm}} = \frac{{{\mathrm{min}}\left( {e,f} \right)}}{{d + {\mathrm{min}}\left( {e,f} \right)}}$, respectively. Given that nestedness and turnover are complementary elements of dissimilarity, i.e., $\mathrm {TD}_{\mathrm {DIS}}|\mathrm {FD}_{\mathrm {DIS}} = \mathrm {TD}_{\mathrm {NES}}|\mathrm {FD}_{\mathrm {NES}} + \mathrm {TD}_{\mathrm {TUR}}|\mathrm {FD}_{\mathrm {TUR}}$, we further quantified the contribution of TD_NES_|FD_NES_ (TD_NESc_|FD_NESc_) and TD_TUR_|FD_TUR_ (TD_TURc_|FD_TURc_) to TD_DIS_|FD_DIS_ as

$$\mathrm {TD}_{\mathrm {NESc}}|\mathrm {FD}_{\mathrm {NESc}} = \frac{{\mathrm {TD}_{\mathrm {NES}}|\mathrm {FD}_{\mathrm {NES}}}}{{\mathrm {TD}_{\mathrm {DIS}}|\mathrm {FD}_{\mathrm {DIS}}}}$$ and $$\mathrm {TD}_{\mathrm {TURc}}|\mathrm {FD}_{\mathrm {TURc}} = \frac{{\mathrm {TD}_{\mathrm {TUR}}|\mathrm {FD}_{\mathrm {TUR}}}}{{\mathrm {TD}_{\mathrm {DIS}}|\mathrm {FD}_{\mathrm {DIS}}}}$$.

For each spatial scale, we assessed changes in dissimilarity, nestedness, turnover, and contributions of nestedness and turnover to dissimilarity across time. As for change in α diversity, we used for this the slope of the long-term relationship between TD_DIS_|FD_DIS_ and time (TD_ΔDIS_|FD_ΔDIS_), TD_NES_|FD_NES_ and time (TD_ΔNES_|FD_ΔNES_), TD_TUR_|FD_TUR_ and time (TD_ΔTUR_|FD_ΔTUR_), TD_NESc_|FD_NESc_ and time (TD_ΔNESc_|FD_ΔNESc_), and TD_TURc_|FD_TURc_ and time (TD_ΔTURc_|FD_ΔTURc_). We also estimated slopes of the long-term relationship between colonizations (TD_COL_|FD_COL_) and extinctions (TD_EXT_|FD_EXT_) (i.e., *b*, *c*, *e*, and *f* components in equations given above) and time (TD_ΔCOL_|FD_ΔCOL_ and TD_ΔEXT_|FD_ΔEXT_). To estimate slopes of long-term relationships of all diversity change metrics across time, we fit mixed effects with a function lme in package nlme (https://cran.r-project.org/web/packages/nlme/index.html). Note that while species richness is usually captured as count data and then best fitted using a Poisson distribution, our estimates of taxonomic diversity were detection-corrected and no longer integer values. The metrics of avian diversity change in our analysis were roughly normally distributed (Supplementary Fig. 11) and we therefore fitted all models using a normal distribution. To account for the variation in temporal trends across grid cells, we included a random effect on the grid cell. To address temporal autocorrelation, we fit an autoregressive-moving average process using a correlation structure specified by the autocorrelation argument corARMA in all models^76^. All models were run in the statistical program R 3.2.3 (https://www.r-project.org/).

### Temporal change in trait space

In addition to quantifying scale dependence of changes in TD and FD, we also assessed the interaction between scale and temporal change for specific parts of avian trait space. To separate trait groups, or guilds, we used dietary niche (i.e., proportions of different types of food in species’ diet—invertebrates, vertebrates, carrion, fresh fruits, nectar and pollen, seeds, and other plant materials), foraging niche (i.e., the proportional use of each of seven niches—water below surface, water around surface, terrestrial ground level, understory, mid canopy, upper canopy, and aerial), and body mass. For dietary and foraging niche, we quantified the relative proportion (i.e., prevalence) of each diet and foraging niche for each assemblage, year, and spatial grain combination. The relative proportion was weighted by the probability of species occurrence, *ψ*_*i*_, to account for the contribution of each species to the functional trait space. Mean body mass of all species comprising the assemblage was also derived, with all body mass values being weighted by the probability of a given species occurrence.

We quantified temporal change in trait space in a similar manner to changes in TD and FD. That is, we assessed temporal changes in mean body mass of the assemblage and temporal changes in the relative proportion of each trait in the assemblage (for all components of dietary and foraging niche) by fitting the slope of the long-term relationship between the respective trait and time—this is equivalent to TD_Δ_ and FD_Δ_.

All slopes were fitted with a function lme in package nlme (https://cran.r-project.org/web/packages/nlme/index.html), including a random effect on the grid cell the autocorrelation argument corARMA in all models.

### Null models

FD is often closely associated with TD. We therefore generated a null model expectation for each grid cell at each and spatial scale to evaluate deviations of observed FD_Δ_ from those expected given TD_Δ_. We developed expected FD for each grid cell and each time period by randomly reshuffling values of probabilities of occurrence across the subset of species of the given assemblage^50^, thus keeping grid cell-level TD constant. We then computed change in expected FD by calculating the slope of the long-term relationship between the expected FD and time (FD_ΔEXP_). The reshuffling for both null models was performed 100 times. We ranked the observed FD_Δ_against FD_ΔEXP_ and calculated the *p* value to indicate the statistical significance of the rank. The *p* values of >0.975 indicate that the observed FD_Δ_ is significantly higher than FD_ΔEXP_, *p* values of <0.025 indicate that the observed FD_Δ_ is significantly lower than expected given change in TD. We present these findings in Supplementary Fig. 6.

### Data availability

Data used in this analysis are publically available at https://www.pwrc.usgs.gov/bbs/rawdata. R code is included in the Supplementary Information.

## Electronic supplementary material

Supplementary Information
