# Supplementary material for: Taxonomic and functional diversity change is scale dependent
Source: Nat Commun. 2018 Jul 2;9:2565. doi: 10.1038/s41467-018-04889-z (PMC6028399; doi:10.1038/s41467-018-04889-z)
Supplement: Supplementary file 1 — Supplementary Information [file 41467_2018_4889_MOESM1_ESM.pdf]

Supplementary Information

Title: Taxonomic and functional diversity change is scale dependent

Authors: Marta A. Jarzyna[1*], Walter Jetz[1,2]

Affiliations:

[1]Department of Ecology and Evolutionary Biology, Yale University, New Haven, CT, 06520, USA.

[2]Department of Life Sciences, Imperial College London, Silwood Park Campus, Buckhurst Road, SL5 7PY Ascot, Berks, UK.

*Correspondence to: marta.jarzyna@yale.edu

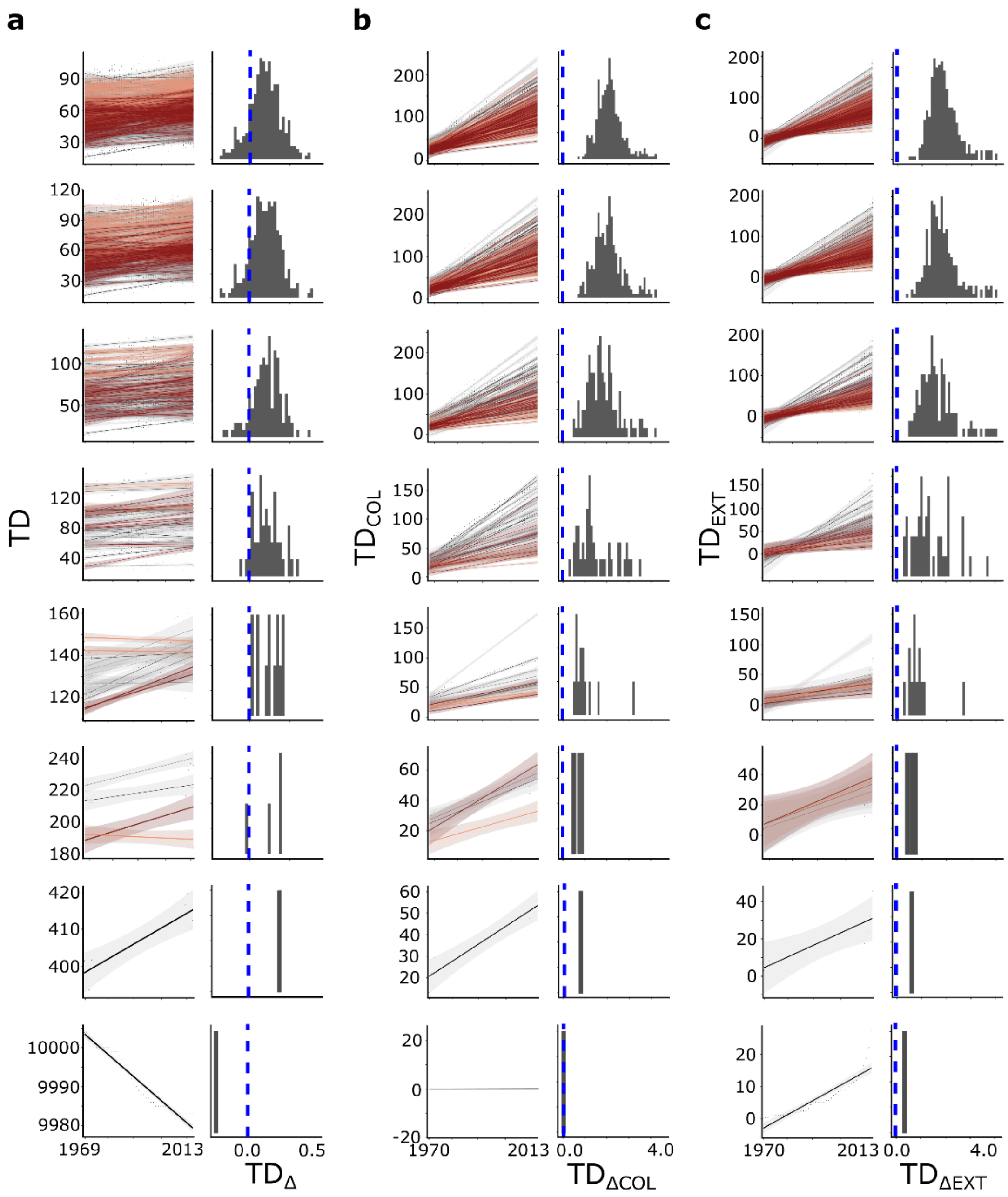

Supplementary Fig. 1. Relationships between (a) taxonomic diversity (TD) and time (TD_{Δ}), (b) the number of colonizations (TD_{COL}) and time ($TD_{\Delta COL}$), and (c) the number of extinctions (TD_{EXT}) and time ($TD_{\Delta EXT}$) across eight spatial scales (from top to bottom: 50 km, 100 km, 200 km, 400 km, 800 km, 1600 km, the continental US, and the globe). Histograms show the distribution of TD_{Δ}, $TD_{\Delta COL}$, and $TD_{\Delta EXT}$; the blue dashed line shows zero. For the continent-

wide analysis, bird occurrence records were obtained from the North American Breeding Bird Survey (1969-2013). We included 494 species, excluding nocturnal, crepuscular, and pelagic species. For the global analysis, we used data of global extinctions from [1].

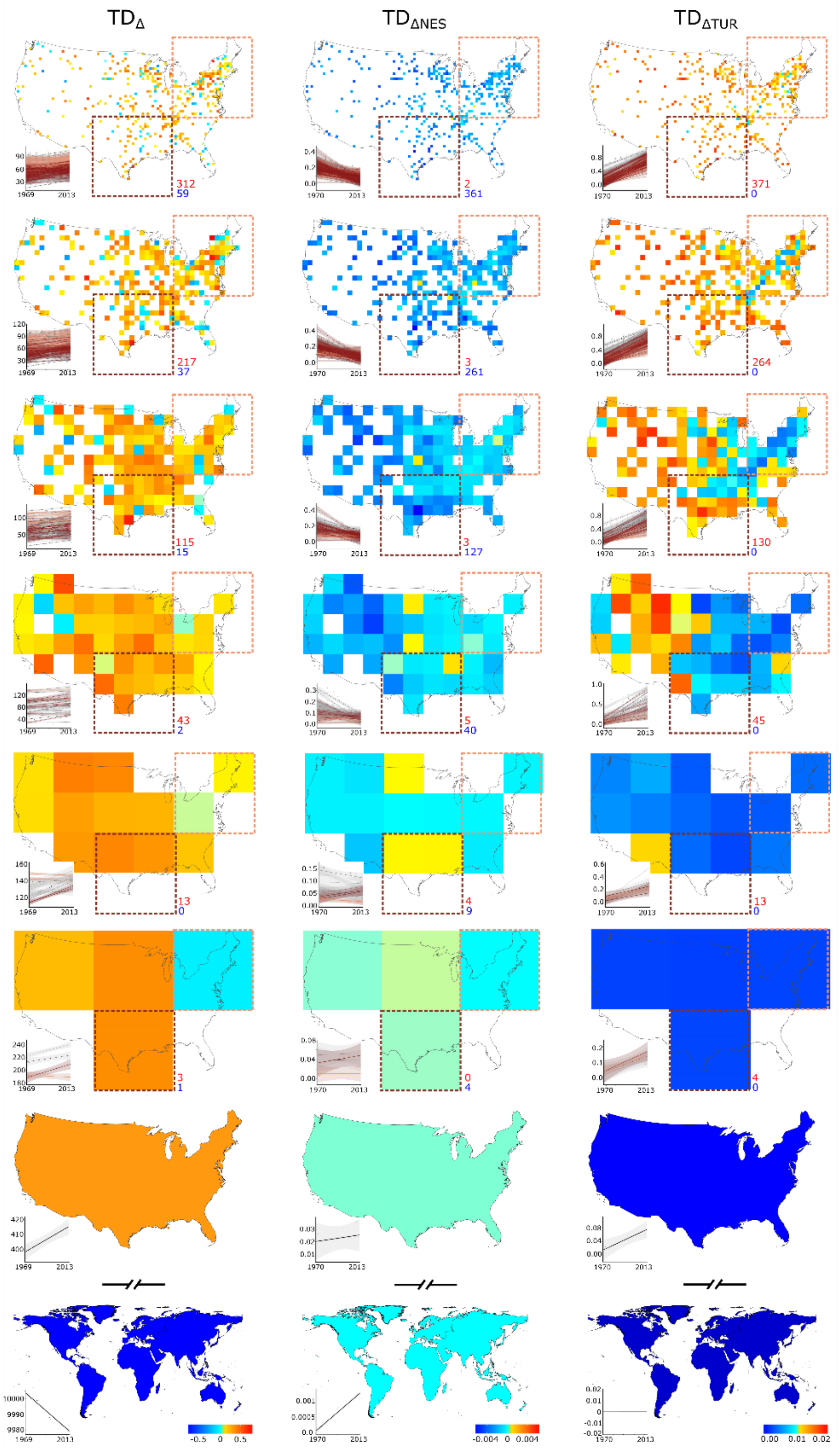

Supplementary Fig. 2. Spatial variation in change in avian diversity across different spatial scales (from top to bottom—50 km, 100 km, 200 km, 400 km, 800 km, 1600 km, the continental US, and the globe). Change was assessed as the fitted slopes between time and taxonomic

diversity (TD_{Δ}, left panel), temporal nestedness of taxonomic diversity ($TD_{\Delta NES}$, middle panel), and temporal turnover of taxonomic diversity ($TD_{\Delta TUR}$, right panel). Fitted positive and negative slopes indicate increases and declines in their respective measure of change. Red and blue numbers indicate the number of grid cells for which these trends were positive and negative, respectively. Insets show the fitted slopes between the respective measure of change (y axis) and time (x axis; calendar year) for the cell inside the brown (brown lines) or beige (beige lines) quadratic regions. For data, see Supplementary Fig. 1.

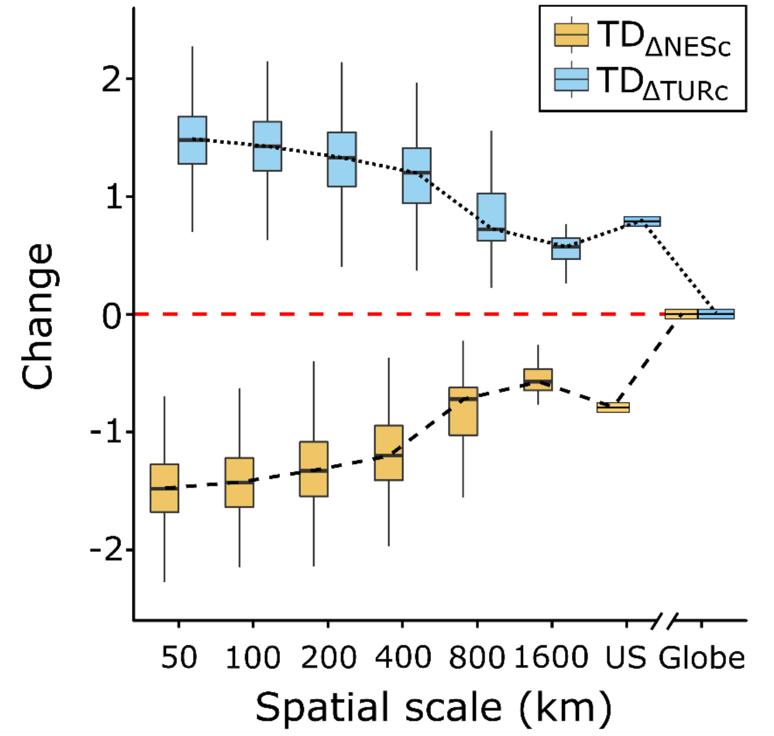

Supplementary Fig. 3. Changes in the contribution of temporal nestedness and turnover of taxonomic diversity (TD_{NESc} and TD_{TURc}, respectively) to temporal dissimilarity (TD_{DIS}) are scale invariant as evidenced by the fitted slopes between time and TD_{NESc} ($TD_{\Delta NESc}$) and TD_{TURc} ($TD_{\Delta TURc}$). Boxes represent the 25th and 75th percentiles, lines within the boxes represent the 50th percentile (median), and whiskers represent 2.5^{th} and 97.5^{th} percentiles. For data, see Supplementary Fig. 1.

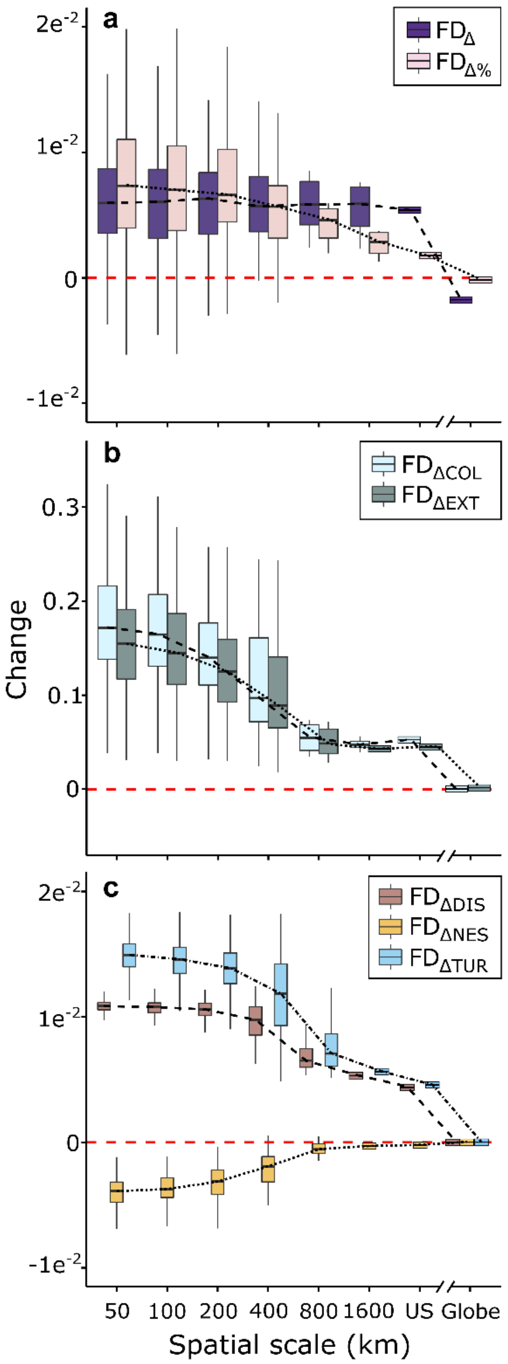

Supplementary Fig. 4. Changes in the functional diversity (FD) are scale dependent as evidenced by the fitted slopes between time and (a) FD (FD_{Δ}) and relative change in FD ($FD_{\Delta\%}$) and (b) functional diversity of colonizations and extinctions ($FD_{\Delta COL}$ and $FD_{\Delta EXT}$, respectively) at eight spatial scales. (c) Changes in temporal β diversity of FD given by the fitted slopes

between temporal dissimilarity of functional diversity and time ($FD_{\Delta DIS}$), also show strong scale dependence, as do the components of $FD_{\Delta DIS}$, temporal nestedness and turnover of functional diversity ($FD_{\Delta NES}$ and $FD_{\Delta TUR}$, respectively). Compilation of function-relevant traits was obtained from [2]. For other details see Supplementary Fig. 1.

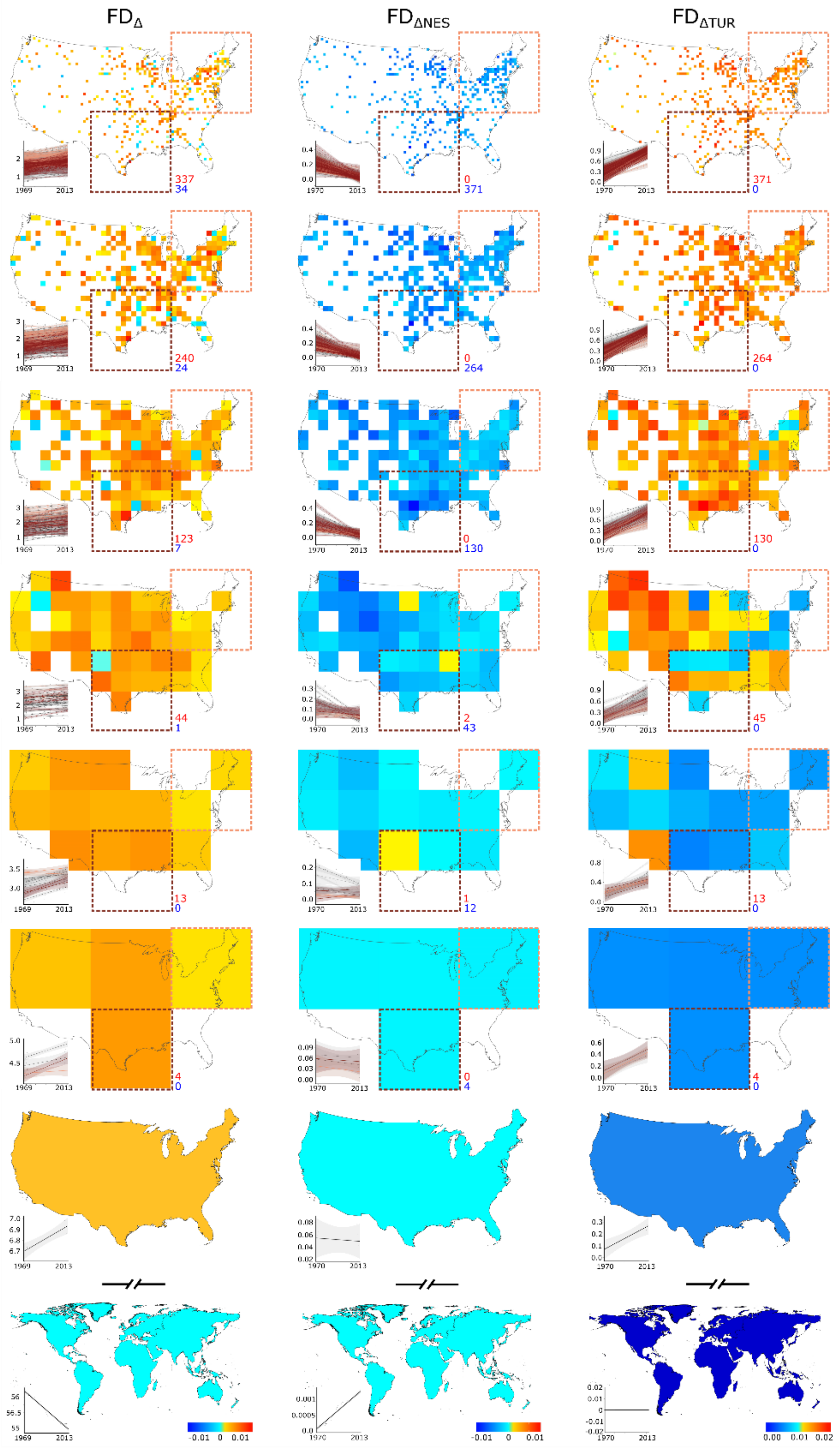

Supplementary Fig. 5. Spatial variation in change in avian functional diversity across different spatial scales (from top to bottom—50 km, 100 km, 200 km, 400 km, 800 km, 1600 km, the continental US, and the globe). Change was assessed as the fitted slopes between time and

functional diversity (FD_{Δ}, left panel), temporal nestedness of functional diversity ($FD_{\Delta NES}$, middle panel), and temporal turnover of functional diversity ($FD_{\Delta TUR}$, right panel). Fitted positive and negative slopes indicate increases and declines in their respective measure of change. Red and blue numbers indicate the number of grid cells for which these trends were positive and negative, respectively. Insets show the fitted slopes between the respective measure of change (y axis) and time (x axis; calendar year) for the cell inside the brown (brown lines) or beige (beige lines) quadratic regions. For data, see Supplementary Fig. 4.

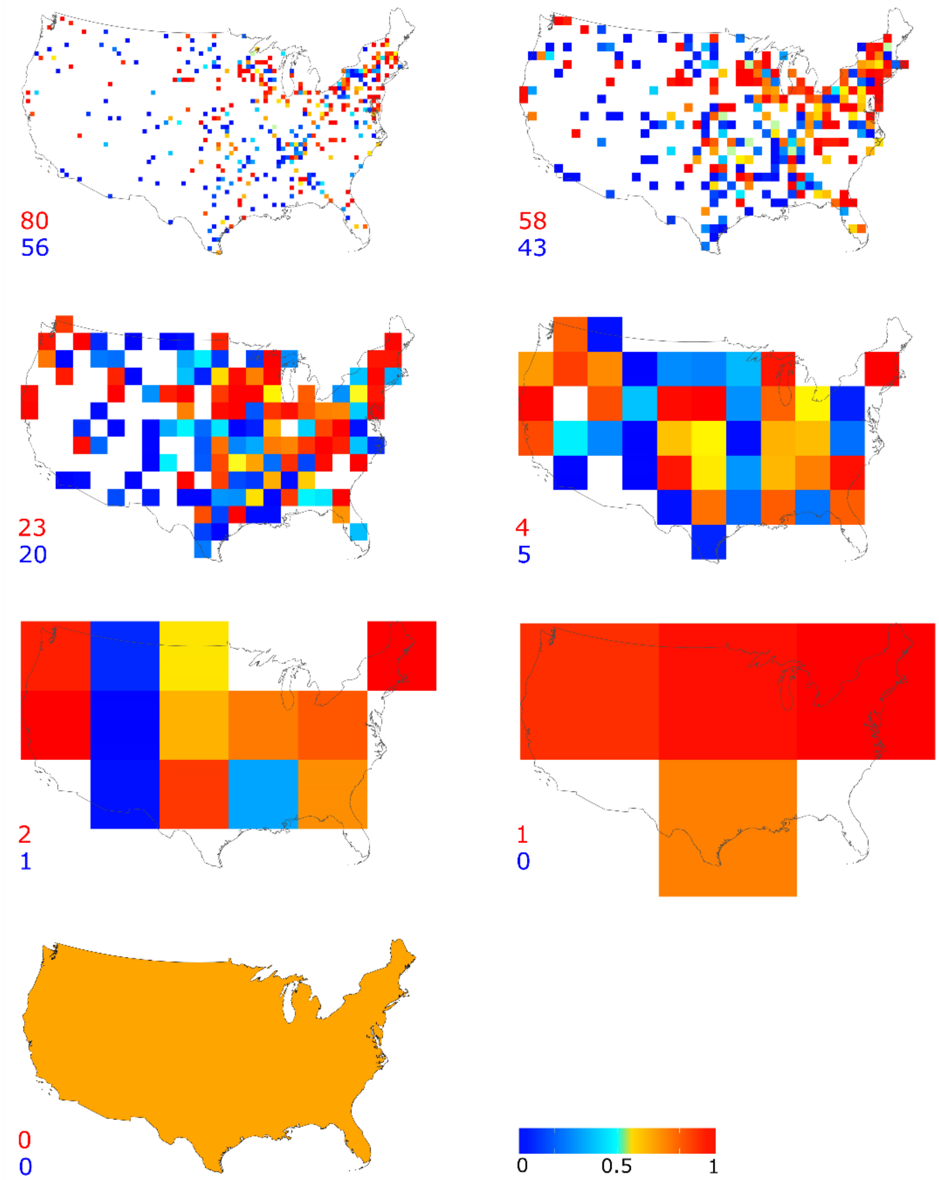

Supplementary Fig. 6. Spatial distributions of the p-values resulting from ranking the observed fitted slopes between functional diversity and time (FD_{Δ}) against the expected fitted slopes between functional diversity and time ($FD_{\Delta EXP}$) given the observed fitted slopes between taxonomic diversity and time (TD_{Δ}) for 50 km, 100 km, 200 km, 400 km, 800 km, 1600 km, and the continental US. P-values >0.975 and <0.025 indicate that FD_{Δ} was significantly higher and

lower, respectively, than $FD_{\Delta EXP}$. Red and blue numbers indicate the number of grid cells for which p-values were >0.975 (red) and <0.025 (blue).

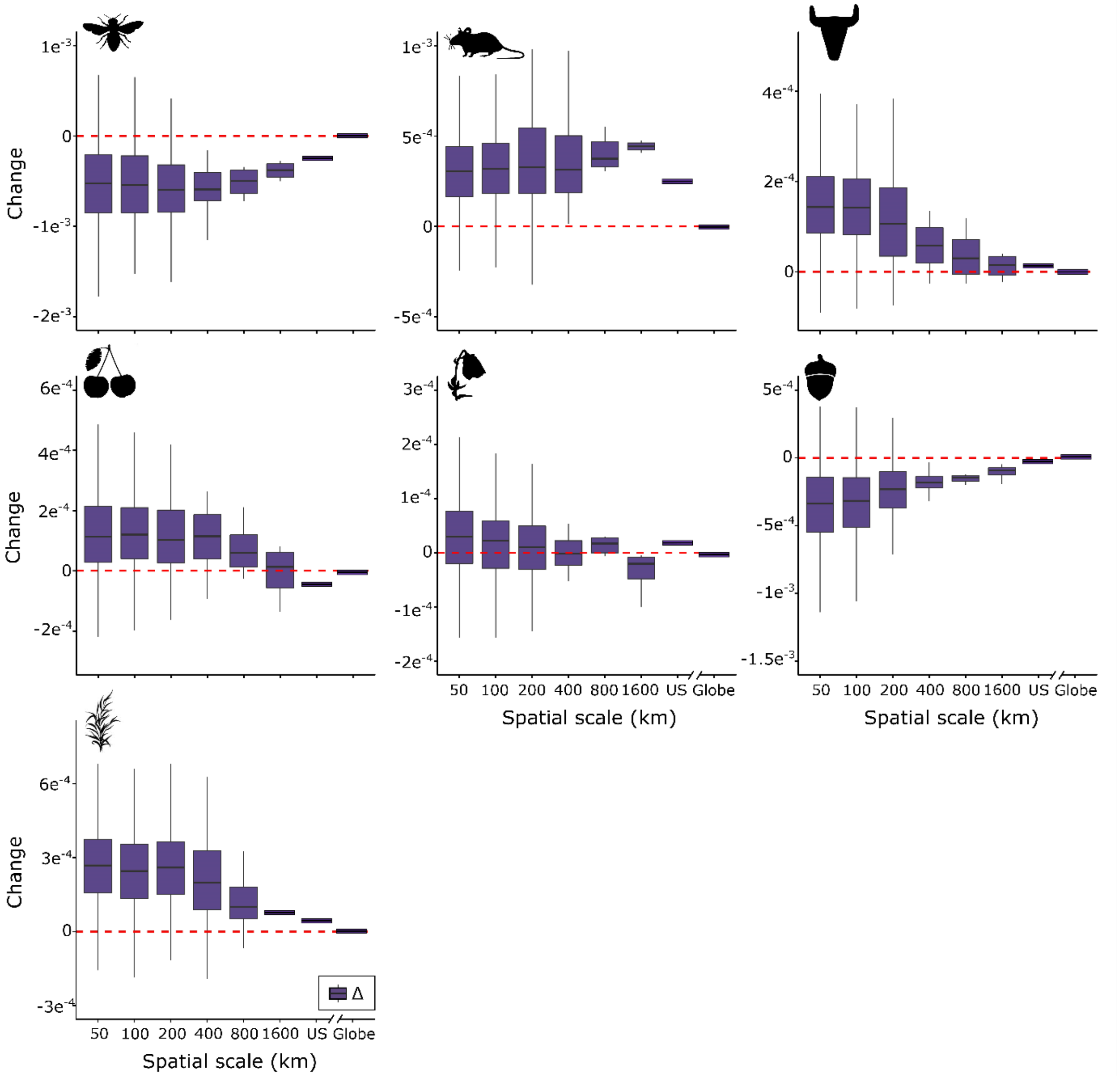

Supplementary Fig. 7. Scale dependence of changes in the proportion of (top row) insectivorous, vertebrate, scavenging, (middle row) frugivorous, nectarivorous, granivorous, and (bottom row) plant matter diets are scale dependent as shown by the fitted slopes between the proportion of a given diet and time (Δ). For data, see Supplementary Figs. 1 and 4.

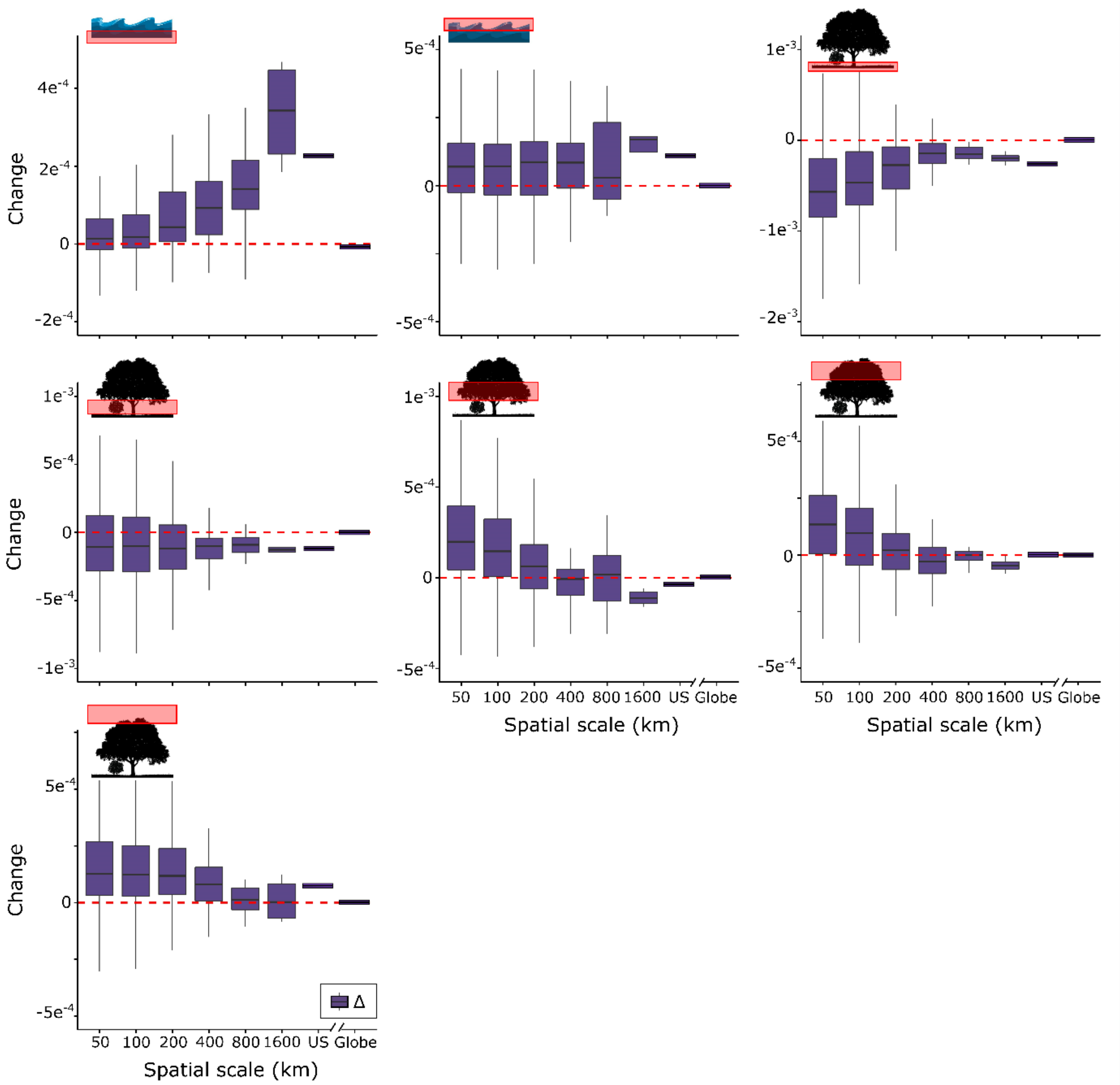

Supplementary Fig. 8. Scale dependence of changes in the proportion of (top row) water below surface, water around surface, ground, (middle row) understory, mid canopy, upper canopy, and (bottom row) aerial foraging heights are scale dependent as shown by the fitted slopes between the proportion of a given foraging height and time (Δ). For data, see Supplementary Figs. 1 and 4.

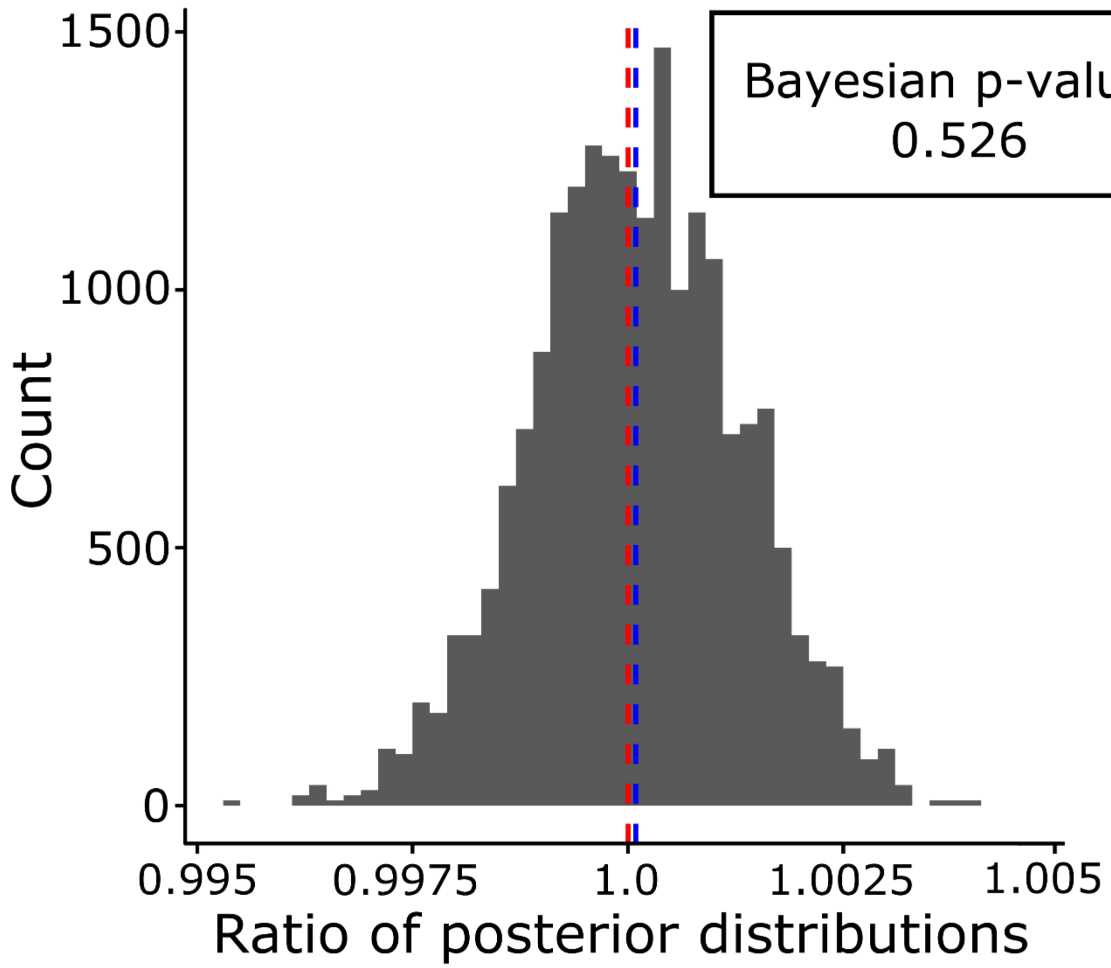

Supplementary Fig. 9. Model fit was evaluated using posterior predictive checking, here shown for the occupancy model for year 2013. Histogram shows the distribution of the values obtained by taking the ratio of the posterior distribution of the fit statistic to the posterior distribution of that statistic for hypothetical perfect data sets for which the model is known to be correct. Blue dashed line shows the mean value of the distribution (1.00009), with values of the ratio close to 1 (red dashed line) indicating good model fit. The Bayesian p-value for this model is 0.526, with values close to 0.5 indicative of good model fit.

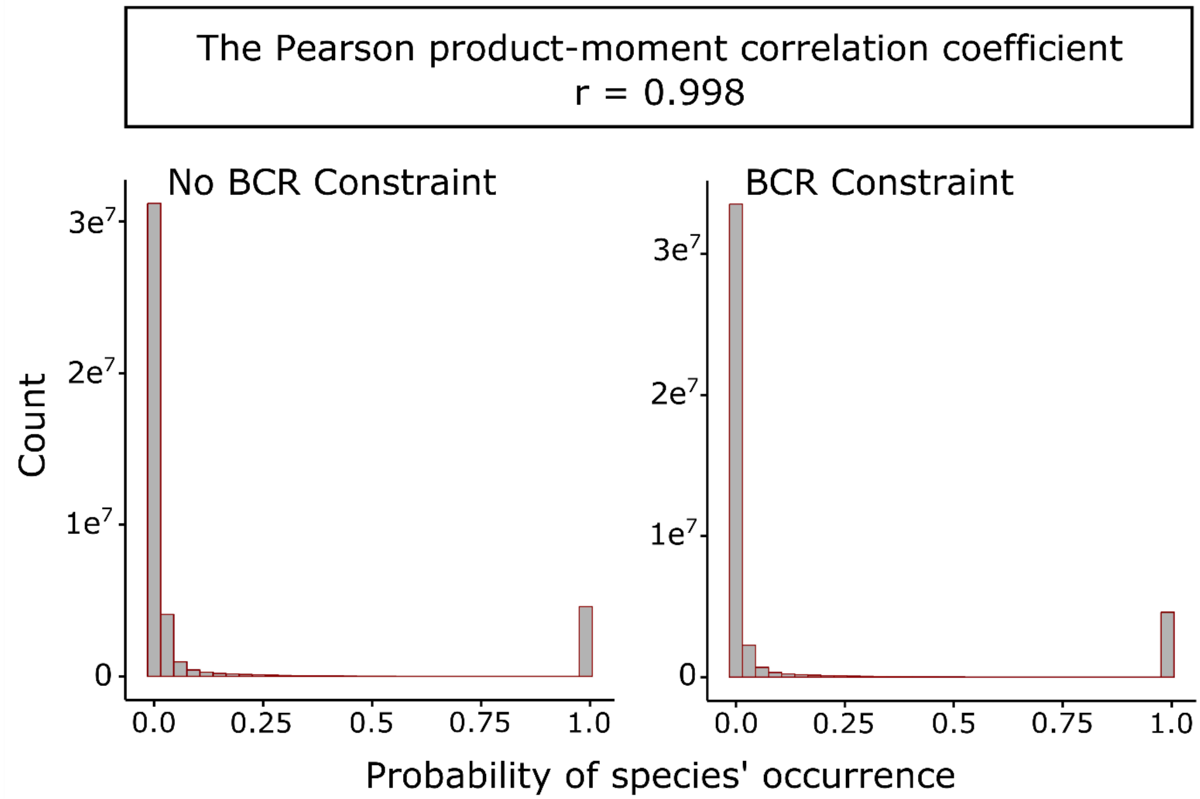

Supplementary Fig. 10. Distribution of probabilities of occurrence for all species resulting from occupancy models before (left panel) and after (right panel) imposing the Bird Conservation Region (BCR) constraint. Following the BCR constraint, only species detected within a given BCR in that year could have $\psi_{i,j}$ >0 at a route located within that BCR. The constraint was imposed in order to avoid instances where probability of occurrence $\psi_{i,j}$ >0 for species that are unlikely to be present given their ecological constraints.

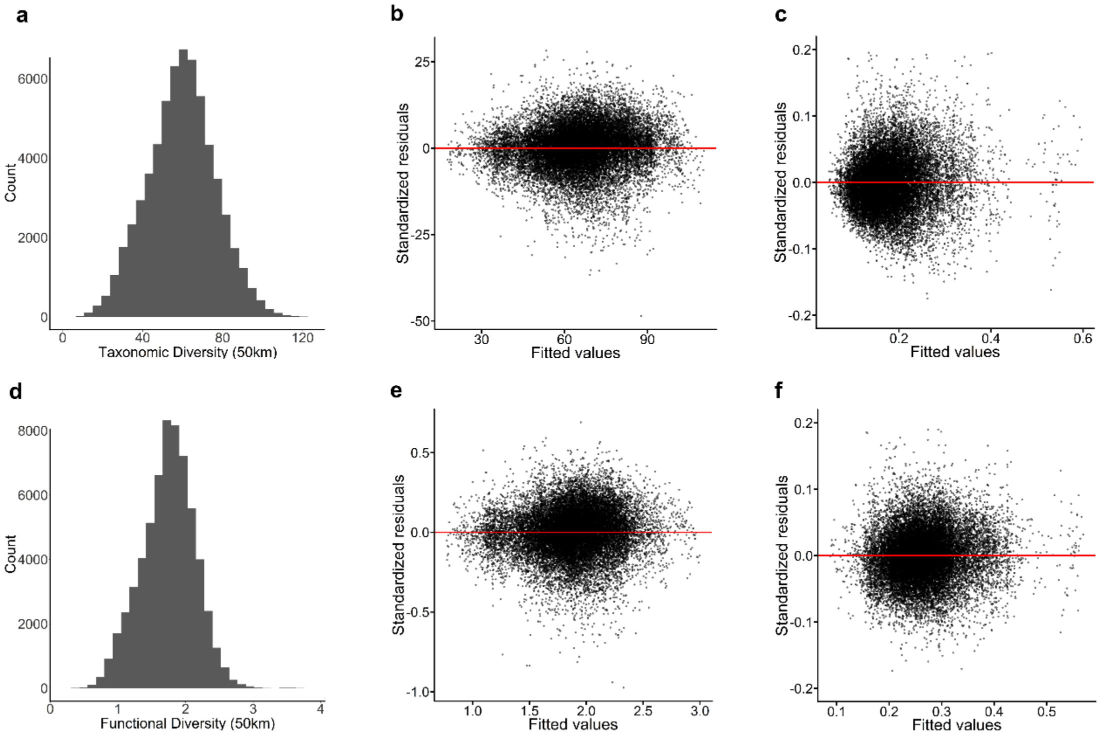

Supplementary Fig. 11. Histograms of (a) taxonomic (TD) and (d) functional (FD) diversity for an example spatial scale (here, 50km) indicate values follow an approximately normal distribution. This is affirmed by scatterplots of standardized residuals and fitted values resulting from fitting mixed effects models with a normal distribution for (b) TD, (c) temporal turnover in TD (TD_{TUR}), (e) FD, (f) temporal turnover in FD (FD_{TUR}) at the spatial scale of 50km.

Supplementary Table 1. Extinct, Extinct in the Wild, and Critically Endangered (Possibly Extinct) avian species since 1969 [1].

Common Name	Scientific Name	Estimated extinction date	Species status	Trait information source
Alagoas Curassow	*Mitu mitu*	1988	Extinct in the Wild	[2]
Alaotra Grebe	*Tachybaptus rufolavatus*	1997	Extinct	Morphologically similar species T. pelzelnii (http://www.iucnredlist.org/) retrieved from [2]
Atitlan Grebe	*Podilymbus gigas*	1985	Extinct	Sister species *P. podiceps* [3] retrieved from [2]
Colombian Grebe	*Podiceps andinus*	1979	Extinct	Sister species *P. nigricollis* [4] retrieved from [2]
Bar-winged Rail	*Nesoclopeus poecilopterus*	1973	Extinct	Sister species *N. woodfordi* [5] retrieved from [2]
Guam Rail	*Gallirallus owstoni*	1987	Extinct in the Wild	[2]
Socorro Dove	*Zenaida graysoni*	1972	Extinct in the Wild	[2]
North Island Piopio	*Turnagra tanagra*	1970	Extinct	Morphologically similar genus *Sphecotheres* [6] (mean trait values) retrieved from [2]
Eiao Monarch	*Pomarea fluxa*	1982	Extinct	Sister species *P. iphis* (http://eol.org/) retrieved from [2]
Ua Pou Monarch	*Pomarea mira*	1987	Critically Endangered (Possibly Extinct)	Sister species *P. mendozae* [7] retrieved from [2]
Guam Flycatcher	*Myiagra freycineti*	1983	Extinct	Sister species *M. oceanica* (http://avibase.bsc-eoc.org) retrieved from [2]
Hawaiian Crow	*Corvus hawaiiensis*	2003	Extinct in the Wild	[2]
Kauai Oo	*Moho braccatus*	1993	Extinct	Morphologically-similar family *Meliphagidae* (mean trait values) retrieved from [2]
Bishop's Oo	*Moho bishopi*	1991	Extinct	Morphologically-similar family *Meliphagidae* (mean trait values) retrieved from [2]
Aldabra Warbler	*Nesillas aldabrana*	1985	Extinct	Mean trait values for genus *Nesillas* retrieved from [2]
White-chested White-eye	*Zosterops albogularis*	1995	Critically Endangered (Possibly Extinct)	[2]
Kamao	*Myadestes myadestinus*	1990	Extinct	Sister species *M. palmeri* (http://www.iucnredlist.org) retrieved from [2]
Ou	*Psittirostra psittacea*	1989	Critically Endangered	[2]

			(Possibly Extinct)	
Kakawahie	*Paroreomyza flammea*	1970	Extinct	Sister species *P. montana* [8] retrieved from [2]
Oahu Alauahio	*Paroreomyza maculata*	1985	Critically Endangered (Possibly Extinct)	[2]
Poo-uli	*Melamprosops phaeosoma*	2004	Critically Endangered (Possibly Extinct)	[2]

Supplementary References

1 Szabo, J. K., Khwaja, N., Garnett, S. T. & Butchart, S. H. M. Global Patterns and Drivers of Avian Extinctions at the Species and Subspecies Level. *PLOS ONE* **7**, e47080, doi:10.1371/journal.pone.0047080 (2012).

2 Wilman, H. *et al.* EltonTraits 1.0: Species-level foraging attributes of the world's birds and mammals. *Ecology* **95**, 2027-2027, doi:10.1890/13-1917.1 (2014).

3 Livezey, B. C. FLIGHTLESSNESS IN GREBES (AVES, PODICIPEDIDAE): ITS INDEPENDENT EVOLUTION IN THREE GENERA. *Evolution* **43**, 29-54, doi:10.1111/j.1558-5646.1989.tb04205.x (1989).

4 Llimona, F., del Hoyo, J., Christie, D.A., Jutglar, F. & Kirwan, G.M. in *Handbook of the Birds of the World Alive.* (ed J. del Hoyo, Elliott, A., Sargatal, J., Christie, D.A. & de Juana, E.) (Lynx Edicions, 2017).

5 Taylor, B. in *Handbook of the Birds of the World Alive.* (ed J. del Hoyo, Elliott, A., Sargatal, J., Christie, D.A. & de Juana, E.) (Lynx Edicions, 2017).

6 Johansson, U. S., Pasquet, E. & Irestedt, M. The New Zealand Thrush: An Extinct Oriole. *PLOS ONE* **6**, e24317, doi:10.1371/journal.pone.0024317 (2011).

7 del Hoyo, J. C., N. in *Handbook of the Birds of the World Alive* (ed J. del Hoyo, Elliott, A., Sargatal, J., Christie, D.A. & de Juana, E.) (Lynx Edicions, 2017).

8 Tokita, M., Yano, W., James, H. F. & Abzhanov, A. Cranial shape evolution in adaptive radiations of birds: comparative morphometrics of Darwin's finches and Hawaiian honeycreepers. *Philosophical Transactions of the Royal Society B: Biological Sciences* **372**, doi:10.1098/rstb.2015.0481 (2017).

Supplementary Note 1: Supplementary R code

R code for occupancy model

```
#-----------------------------------------------------------------------
#Multi-species occupancy model using R and JAGS
#Modeling individual species occupancy given occurrence and detection prob
```

```
#Jarzyna & Jetz, Nature Communications, 2018
#-----------------------------------------------------------------------
require(coda)
require(rjags)

########################read in data and covariates
Xdata <- as.matrix(read.table("2013_SEG.txt", header=TRUE))
occvar <- read.table("2013_ELEV_NArem.txt", header=TRUE)

nsite=nrow(occvar) #nsite is the number of sampled points
nspec=ncol(Xdata)-1 #nspec is the number of observed species
nrep_no=5 #nrep is a vector of length nsite indicating the number of reps at each nsite

encount <- Xdata[,2:ncol(Xdata)]
X <- array(encount, dim = c(nsite, nrep_no, nspec))
nrep <- rep(5,nsite) #number of repeated visits

#occupancy covariates
elevsc <- scale(occvar[,2])


########################Load all the data: detection array and covariate information
sp.data = list(nspec=nspec, nsite=nsite, nrep=nrep, X=X, elev=elevsc)

###Specify the parameters to be monitored
sp.params = list("Z", "occ_sp", "p.fit", "p.fitnew")
#Z matrix will store true prob of occurrence
#occ_sp will store mean occupancy across all sites for all species
#p.fit and p.fitnew are required for Bayesian p value

sp.params <- as.character(sp.params)

########################Specify the initial values
Zobs <- as.matrix(read.table("2013_OBS.txt", header=TRUE))

#Set 1 - this set of initial values has proven effective, so I'll be using these
sp.inits = function() {
psi.meanGuess = runif(1,0.001,0.99)
list(psi.mean=psi.meanGuess, theta.mean=runif(1,0.001,0.99),
u=rnorm(nspec), v=rnorm(nspec),
Z = Zobs,
alpha1=rnorm(nspec))
}


########################JAGS model
```

```
cat("
model {

#Prior distributions on the community level occupancy
#and detection covariates
psi.mean ~ dunif(0.001,0.99) #vague prior for the hyperparameter of the community-level
occupancy covariates
a <- log(psi.mean) - log(1-psi.mean)

theta.mean ~ dunif(0.001,0.99) #vague prior for the hyperparameter of the community-level
detection covariates
b <- log(theta.mean) - log(1-theta.mean)

mu.alpha1 ~ dnorm(0, 0.01)

tau1 ~ dgamma(10,1)
tau2 ~ dgamma(10,1)

tau.alpha1 ~ dgamma(10,1)#Zipkin's original priors

rho ~ dunif(-0.99,0.99)
var.v <- tau2 /(1.-pow(rho,2))

sigma1 <- 1/sqrt(tau1)
sigma2 <- 1/sqrt(tau2)

for (i in 1:nspec) {

   #Prior distributions for the occupancy and detection covariates for each species
    u[i] ~ dnorm(a, tau1)

    mu.v[i] <- b + (rho*sigma2 /sigma1)*(u[i]-a)
    v[i] ~ dnorm(mu.v[i], var.v)

    alpha1[i] ~ dnorm(mu.alpha1, tau.alpha1)

   #Estimate the occupancy probability (latent Z matrix) for each species
   #at each point (i.e., route or site)
    for (j in 1:nsite) {
      logit(psi[j,i]) <- u[i] + alpha1[i]*elev[j]
            mu.psi[j,i] <- psi[j,i]
      Z[j,i] ~ dbin(psi[j,i], 1)#Z is generally not observed with certainty, instead
                        #we observed data theta[i,j,k] for species i at site j during sampling period k

   #Estimate the species specific detection probability for every rep at each point where the
species occurs (Z=1)
```

```
      for (k in 1:nrep[j]) {
       logit(theta[j,k,i]) <- v[i]
             mu.theta[j,k,i] <- theta[j,k,i]*Z[j,i]
             X[j,k,i] ~ dbin(mu.theta[j,k,i], 1) #X is the 3D array of dependent variable: The detection/non-
                          #detection data is defined in a three dimensional
                          #array X where the first dimension, j, is the point; the second
                          #dimension, k, is the rep; and the last dimension, i, is the species.
       Xnew[j,k,i] ~ dbin(mu.theta[j,k,i], 1) #what is Xnew?

#Create simulated dataset to calculate the Bayesian p-value
d[j,k,i] <-  abs(X[j,k,i] - mu.theta[j,k,i])
dnew[j,k,i] <- abs(Xnew[j,k,i] - mu.theta[j,k,i])
d2[j,k,i] <- pow(d[j,k,i],2)
dnew2[j,k,i] <- pow(dnew[j,k,i],2)
             }
      dsum[j,i] <- sum(d2[j,1:nrep[j],i])
      dnewsum[j,i] <- sum(dnew2[j,1:nrep[j],i])
}
}
#Calculate the discrepancy measure, which is then defined as the mean(p.fit > p.fitnew)
       p.fit <- sum(dsum[1:nsite,1:nspec])
       p.fitnew <- sum(dnewsum[1:nsite,1:nspec])

#} } }
#Estimation of species occupancy (averaged across all the sites)
             for(i in 1:nspec) {
             occ_sp[i] <- sum(Z[1:nsite,i])/nsite
             }
             #End model specification
}
", fill=TRUE, file="Multisp_model.txt")



#######################Run the model
ocmod <- jags.model(file = "Multisp_model.txt", inits = sp.inits, data = sp.data, n.chains = 3)

nburn <- 5000
update(ocmod, n.iter = nburn)
out <- coda.samples(ocmod, n.iter = 20000, variable.names = sp.params, thin=10)
out.mcmc <- as.mcmc(out[[1]])
saveRDS(out.mcmc,file="output_2013.rds")
```
